# Profiling serum cytokines in COVID-19 patients reveals IL-6 and IL-10 are disease severity predictors

**DOI:** 10.1080/22221751.2020.1770129

**Published:** 2020-05-31

**Authors:** Huan Han, Qingfeng Ma, Cong Li, Rui Liu, Li Zhao, Wei Wang, Pingan Zhang, Xinghui Liu, Guosheng Gao, Fang Liu, Yingan Jiang, Xiaoming Cheng, Chengliang Zhu, Yuchen Xia

**Affiliations:** a Department of Clinical Laboratory, Renmin Hospital of Wuhan University, Wuhan, People’s Republic of China; b Department of Clinical Laboratory, Liyuan Hospital, Tongji Medical College, Huazhong University of Science and Technology, Wuhan, People’s Republic of China; c State Key Laboratory of Virology and Hubei Province Key Laboratory of Allergy and Immunology, School of Basic Medical Sciences, Wuhan University, Wuhan, People’s Republic of China; d Department of Clinical Laboratory, Puai Hospital, Tongji Medical College, Huazhong University of Science and Technology, Wuhan, People’s Republic of China; e Department of Clinical Laboratory, Shanghai Gongli Hospital, the Second Military Medical University, Shanghai, People’s Republic of China; f Department of Clinical Laboratory, HwaMei Hospital, University of Chinese Academy of Sciences, Ningbo, People’s Republic of China; g State Key Laboratory of Virology, College of Life Sciences, Wuhan University, Wuhan, People’s Republic of China; h Department of Infectious Diseases, Renmin Hospital, Wuhan University, Wuhan, People’s Republic of China; i Liver Diseases Branch, National Institute of Diabetes and Digestive and Kidney Diseases, National Institutes of Health, Bethesda, MD, USA

**Keywords:** SARS-CoV-2, COVID-19, cytokine storm, inflammatory cytokines, Interleukin 6, Interleukin 10

## Abstract

Since the outbreak of coronavirus disease 2019 (COVID-19) in Wuhan, China, it has rapidly spread across many other countries. While the majority of patients were considered mild, critically ill patients involving respiratory failure and multiple organ dysfunction syndrome are not uncommon, which could result death. We hypothesized that cytokine storm is associated with severe outcome. We enrolled 102 COVID-19 patients who were admitted to Renmin Hospital (Wuhan, China). All patients were classified into moderate, severe and critical groups according to their symptoms. 45 control samples of healthy volunteers were also included. Inflammatory cytokines and C-Reactive Protein (CRP) profiles of serum samples were analyzed by specific immunoassays. Results showed that COVID-19 patients have higher serum level of cytokines (TNF-α, IFN-γ, IL-2, IL-4, IL-6 and IL-10) and CRP than control individuals. Within COVID-19 patients, serum IL-6 and IL-10 levels are significantly higher in critical group (*n* = 17) than in moderate (*n* = 42) and severe (*n* = 43) group. The levels of IL-10 is positively correlated with CRP amount (*r* = 0.41, *P* < 0.01). Using univariate logistic regression analysis, IL-6 and IL-10 are found to be predictive of disease severity and receiver operating curve analysis could further confirm this result (AUC = 0.841, 0.822 respectively). Our result indicated higher levels of cytokine storm is associated with more severe disease development. Among them, IL-6 and IL-10 can be used as predictors for fast diagnosis of patients with higher risk of disease deterioration. Given the high levels of cytokines induced by SARS-CoV-2, treatment to reduce inflammation-related lung damage is critical.

## Introduction

Coronavirus disease 2019 (COVID-19), which is caused by severe acute respiratory syndrome coronavirus 2 (SARS-CoV-2), was first reported in December, 2019, and the affected countries has grown rapidly and recently WHO has been declared it as a pandemic. Majority of patients with COVID-19 are asymptomatic or experienced mild to severe respiratory illness. However, fatal cases with multi-organ and systemic manifestations like sepsis, septic shock, and multiple organ dysfunction syndromes (MODS) have also been observed [1].

The laboratory study has shown SARS-CoV-2 is cytopathic and this could have caused the first damage to the lung as shown by pathological examination [2]. Accompanied by viral amplification, host immune responses become activated, which is supposed to clear the virus and cure the patients. But why a portion of patients had more severe disease development like MODS is still unknown. We hypothesized cytokine storm plays important role in the pathogenesis of severe cases of COVID-19 [1,3].

Cytokine storms can be triggered by various infectious or non-infectious diseases [4], and cause severe damages to multiple organs. Pathogen infections are recognized by the immune system, which consists of two types of responses: an innate immune response that recognizes pathogen-associated molecular patterns (PAMPs) and an antigen-specific adaptive immune response. In both responses, there are several activated cells of the immune system, which play a key role in establishing the environment of cytokines [5,6]. However, exaggerated, excessive synthesized cytokines lead to an acute, severe systemic inflammatory response known as “cytokine storm”. Several experimental studies and clinical trials suggested that cytokine storm correlated directly with tissue injury and an unfavourable prognosis of severe lung diseases. Until now, the cytokine profile of COVID-19 patient with different disease severity is not clear. The aim of this study is to investigate which cytokines were involved in cytokine storm of COVID-19 and the value of cytokines in diagnosis and treatment of the COVID-19.

## Materials and methods

### Study design

This study was approved by the institutional ethics board of Renmin Hospital of Wuhan University (No.WDRY2020-K066). All patients (*n* = 102) with COVID-19 enrolled in this study were diagnosed with SARS-CoV-2 infection and admitted to Renmin Hospital of Wuhan University (Wuhan, China) between Jan 2020 and Feb 2020. All baseline serum samples were collected immediately after hospital admission and were further divided into moderate (*n* = 42), severe (*n* = 43) and critical (*n* = 17) according to China's novel coronavirus pneumonia diagnosis and treatment guideline (5th edition) [7–10]. Some of them (29 moderate, 23 severe and 14 critical) were enrolled into the longitudinal study, and the serum samples were collected multiple times during hospitalization. The detailed criteria are listed as following:

Moderate group: Fever; respiratory symptoms; imaging finding of pneumonia.

Severe group: patients have any of the following conditions: Respiratory distress, RR ≥30 times / minute; The oxygen saturation (SpO_2_) ≤93% at rest; Oxygen partial pressure (PaO_2_)/oxygen concentration (FiO_2_) in arterial blood ≤300 mmHg; >50% lung imaging progress in the short term within 24–48 h.

Critical group: patients have any of the following conditions: Respiratory failure and mechanical ventilation required; Shock; Combining other organ failure, intensive care unit is needed.

Serum samples collected from 45 healthy volunteers were served as controls.

All individuals included the current study have been confirmed with no infection of other pathogenic microorganisms like, respiratory virus other than SARS-CoV-2, HBV, HCV and HIV.

## Laboratory examination of blood samples

Approximately 3∼5 ml of peripheral blood was obtained with collection tube from the subjects in each group, serum samples were separated by 2000 rpm /20 min centrifugation. Serum cytokines were tested using BD FACSCalibur flow cytometer (BD FACSCalibur, BD Bioscience, CA) and human Th1/Th2 cytokines kit (Ceger, Hangzhou, China) following manufactures’ instructions. Briefly, 25ul of serum sample was mixed with capture antibody coupled beads, and then 25ul fluorescent labelled detection antibodies. The samples were mixed and incubated at room temperature in the dark. After 2.5 h incubation, the beads was washed and resuspended with PBS. A recombinant protein standard for each cytokine that provides an internal control was included. The detection was performed by BD FACSCalibur flow cytometer. The detection range of each cytokine is 2.5 pg/ml to 5000 pg/ml. C-reactive protein (CRP) was tested using i-CHROMA immunofluorescence analyzer and supporting kits (i-CHROMA Reader, Boditech Med Inc, Korean).

## Statistical analysis

SPSS 22.0 statistical software was used for statistical analysis. Count data were analyzed by the *χ*^2^ test, non-normal distribution measurement data were expressed as median (p25, p75) and were analyzed by nonparametric Mann–Whitney U-test or Wilcoxon signed-rank test. Normally distributed measurement data were expressed as the mean ± SD and were analyzed by variance analysis. Correlations between paired data were analyzed using the Spearman rank correlation coefficient. A *P* value < 0.05 indicates statistical significance.

## Results

### Inflammatory makers in COVID-19 patients and controls

The basic information of enrolled individuals was listed in Supplementary Table S1, no significant difference of sex and age were found among the 4 groups (*P* > 0.05). The time of sampling, which indicated as days after patient developed fever, also showed no significant difference among three COVID-19 groups (see Supplementary Table 1).

**Figure 1.**
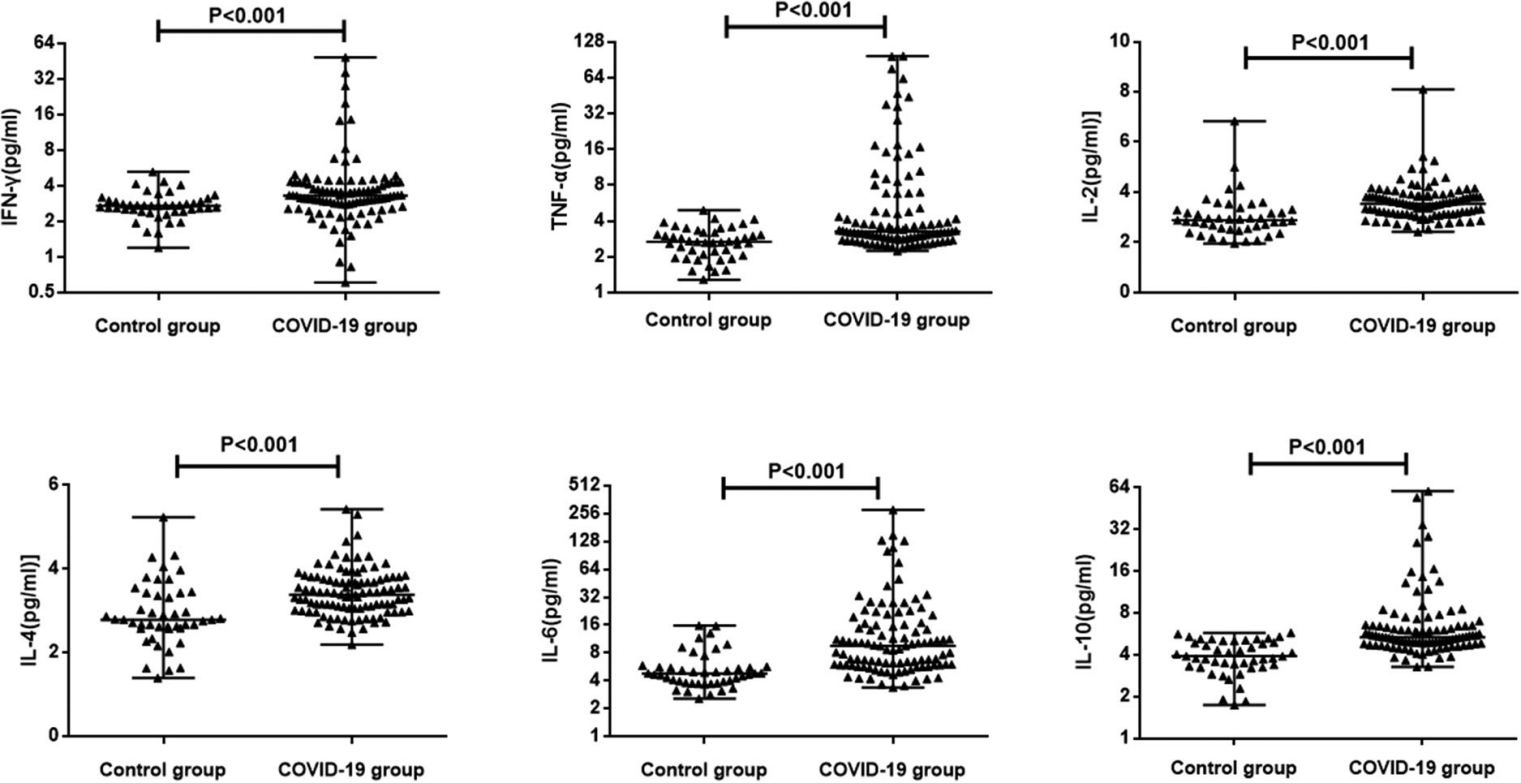
The levels of cytokines in COVID-19 patients and controls. The serum concentration of TNF-α, IFN-γ, IL-2, IL-4, IL-6 and IL-10 from 102 COVID-19 patients and 42 controls were analyzed immediately after hospital admission. Median with range were presented.

We first compared the levels of IFN-γ, TNF-α, IL-2, IL-4, IL-6 and IL-10 in serum samples from control group and COVID-19 patients. As shown in Figure 1, the values of cytokines and CRP were significantly higher in patients with COVID-19 than in healthy controls (*P* < 0.01), suggesting an activation of immune response against SARS-CoV-2 infection. The detailed levels of each group are further shown in Supplementary Table 2. Although most cytokines had a moderate increase of 20% compared to control groups, IL-10 had an 37% increase and IL-6 had 2-fold increase. In consistency with previous report, the median value of CRP in controls is 0.4 mg/L while in COVID-19 patients is 5.56 mg/L, indicating inflammation [11,12].

## Inflammatory makers in COVID-19 patients of different severity

**Figure 2.**
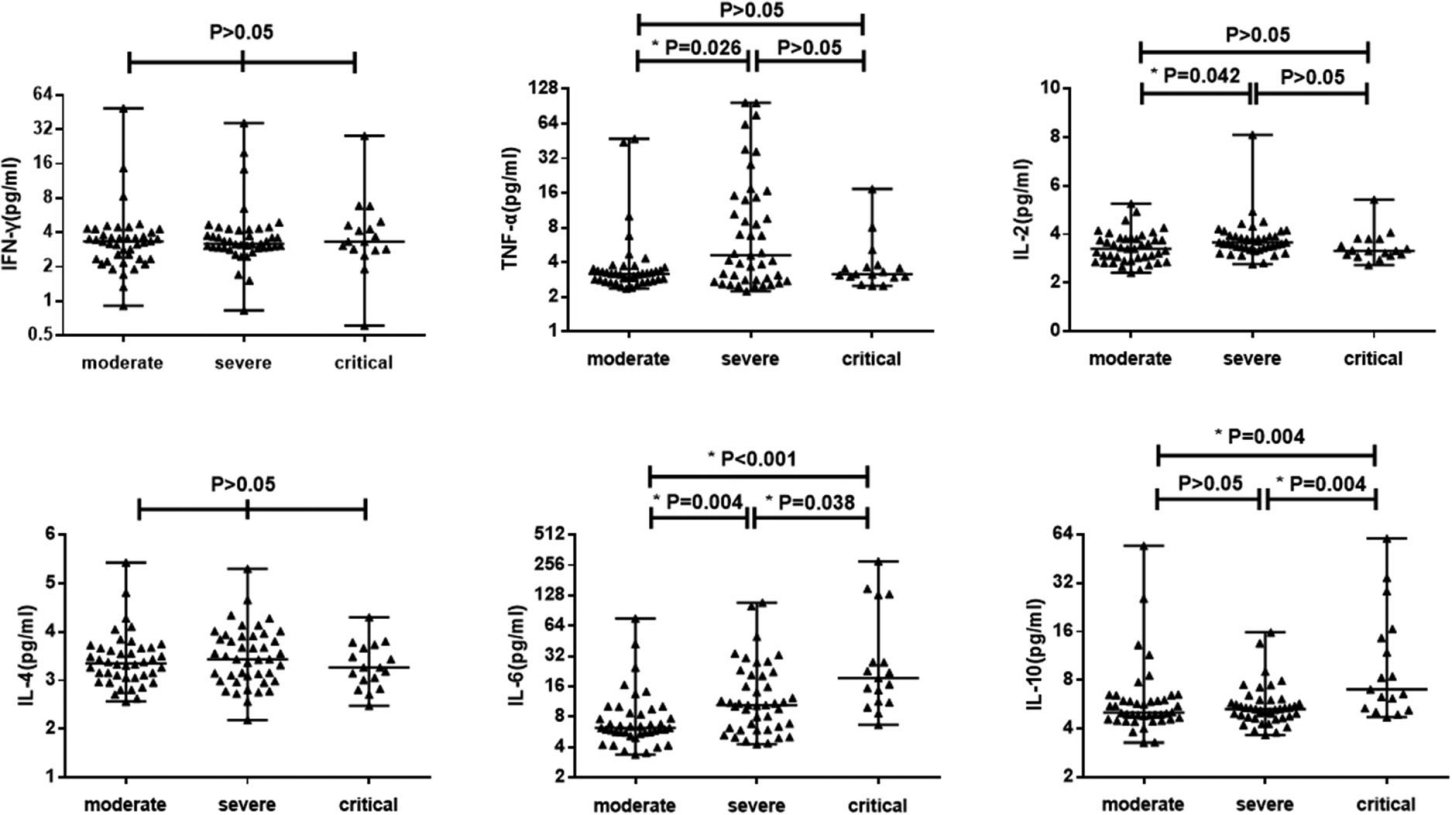
The levels of cytokines in COVID-19 patients with different severity. 102 COVID-19 patients were divided into three groups: moderate, severe and critical. The serum concentration of TNF-α, IFN-γ, IL-2, IL-4, IL-6 and IL-10 were analyzed. Median with range were presented.

To further examine if higher inflammation contributes to disease severity, we next analyzed cytokine profiles and CRP expression of COVID-19 patients sub-grouped based on disease severity (Figure 2). Multivariable comparisons showed that TNF-α, IL-2, IL-6, IL-10 and CRP were statistically different among the different groups (*P* < 0.05), while IFN-γ and IL-4 were not (*P* > 0.05) (Supplementary Table 3). However, only IL-6, IL-10 and CRP showed increased expression along with disease severity but not IL-2 and TNF-α. This trend was more clearly seen when individual’s measurement was plotted (Figure 2). Despite significant differences between critical and moderate groups could be reached by both IL-6 and IL-10, such a significance was not seen for IL-10 between moderate and severe groups, which could be due to limited sample size.

**Figure 3.**
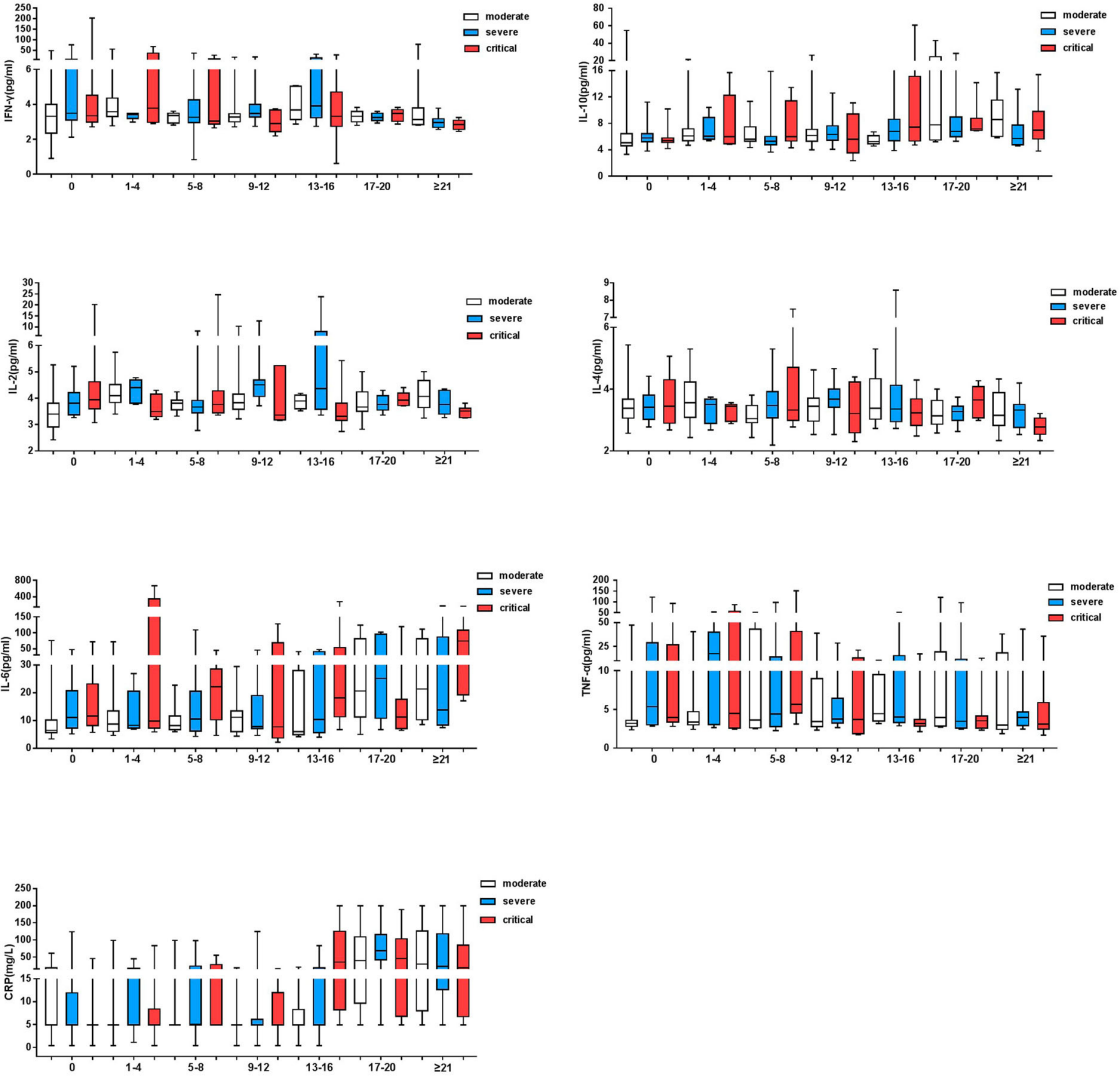
The kinetics of cytokines and CRP in COVID-19 patients during hospitalization. The serum cytokines levels and CRP of moderate, severe and critical patients during hospitalization were presented. The x-axis represents days after admission. Median with range were presented.

To obtain longitudinal changes of different markers in different groups, 29 moderate, 23 severe and 14 critical patients in the above cohort were further monitored during hospitalization. In keeping with previous findings, the cytokines which showed no significant differences (IFN-γ and IL-4) were more stabilized among the majority of patients in each severity group. This contrasted to the dynamic fluctuation observed with TNF-α, IL-2, IL-6, IL-10 and CRP expression, which indicated a timely regulation of those markers along the disease progression (Figure 3 and Supplementary Figure 1). To better view the overall changes of each marker, the serum concentrations of each markers before (baseline level) and after (the last measurement as of March 20, 2020) treatment were compared (Supplementary Figure 2). Of note, with CRP, most patients showed a reduced after treatment (Figure 3 and Supplementary Figure 1 D). In contrast, serum IL-2 were elevated significantly in all three groups (Supplementary Table 4 and Figure 3). In addition, severe group showed elevated IL-4 and reduced IL-6 and TNF-α after treatment.

## Correlations between serum IL-10 with CRP concentration

**Figure 4.**
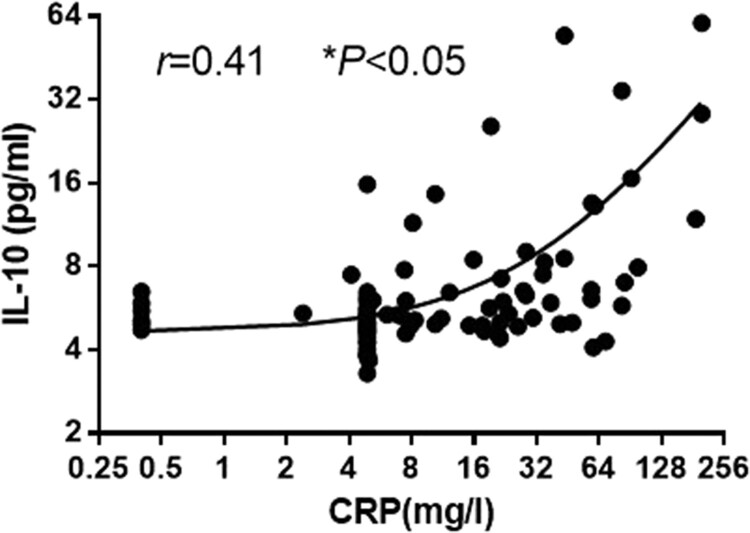
The relationship between CRP and IL-10. Spearman rank correlation analysis was performed to evaluate the correlation of serum IL-10with CRP in the patients with COVID-19.

Previously, small-scale studies have evaluated changes in IL-6 and CRP concentrations for COVID-19 patients [13,14], the correlation between IL-10 and CRP is still unclear. To clarify the relationship between serum IL-10 and CRP, we performed Spearman rank correlation analysis, the results showed that CRP was significantly positively correlated with IL-10 (*r* = 0.41, *P* < 0.01) (Figure 4).

## Potential markers predictive of COVID-19 illness

**Figure 5.**
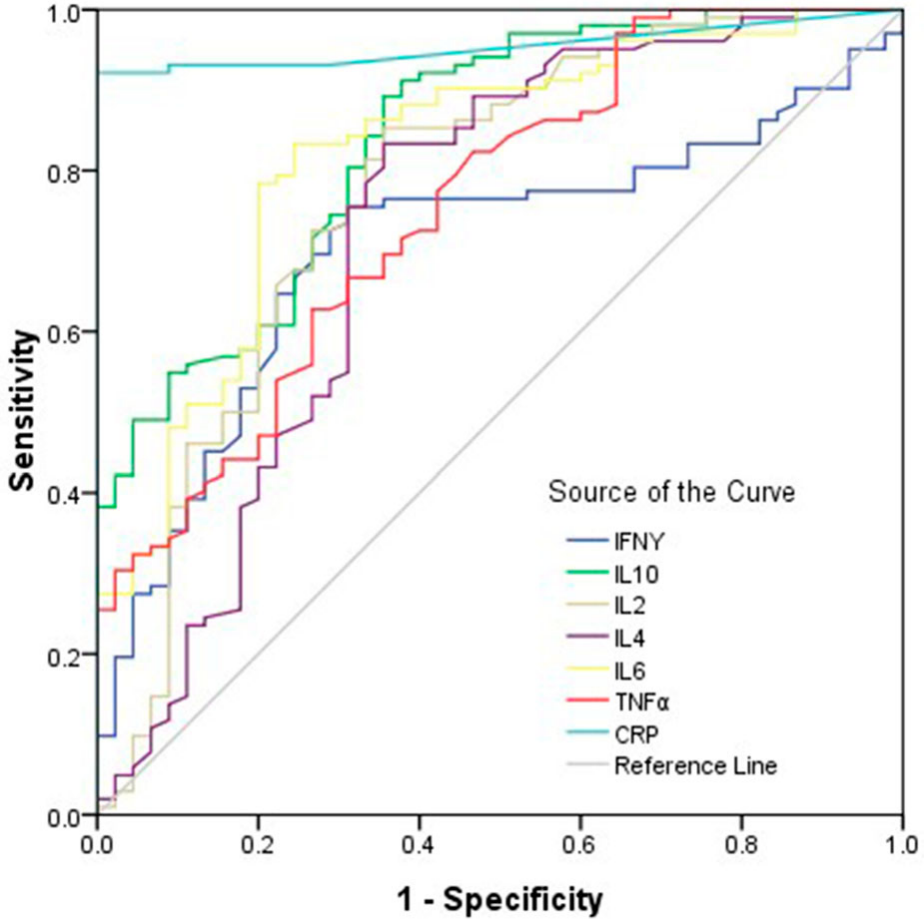
ROC curve of cytokines and CRP. Univariate logistic regression analysis was conducted. Performance of ROC curves of TNF-α, IFN-γ, IL-2, IL-4, IL-6, IL-10 and CRP for predicting COVID-19.

To evaluate the diagnostic value of cytokines in patients with COVID-19, ROC curves were drawn for 102 patients with COVID-19 and healthy controls. For diagnosis of the COVID-19, the area under ROC curve of CRP was the largest among all cytokines; the preoperative CRP concentration of 3.38 mg/L was the optimal cutoff value for predicting COVID-19 (sensitivity = 92.2 (85.1–96.5)%, specificity = 100%), the positive predictive values = 99.0(96.1–100)%, the negative predictive values = 84.9(72.4–93.2)% (Figure 5 and Supplementary Table 5). The combined diagnosis model was established by logistic regression analysis, the area under ROC curve of combined cytokines was 0.99(0.98–1.00), the sensitivity = 93.1(86.4–97.2)%, the specificity = 97.2(85.4–99.5)%, the positive predictive values = 99.0(94.3–99.8)%, the negative predictive values = 83.3(68.6–93.0)% (Figure 5 and Supplementary Table 5).

## Potential markers of diagnostic value to distinguish severe and critical COVID-19

**Figure 6.**
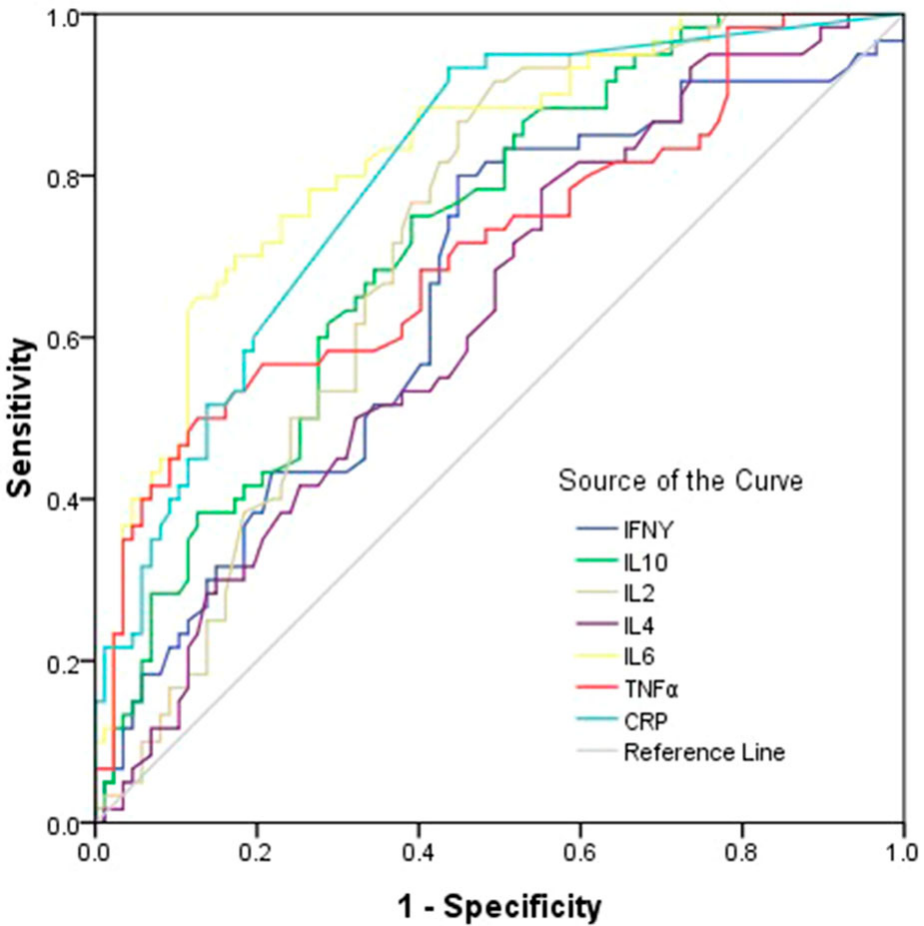
ROC curve for diagnosis of severe and critical patients with COVID-19. Univariate logistic regression analysis was used to identify the severe and critical patients from controls and moderate COVID-19 patients. Performance of ROC curves of TNF-α, IFN-γ, IL-2, IL-4, IL-6, IL-10 and CRP for predicting severe and critical COVID-19 patients.

To evaluate the diagnostic value of cytokines in patients with severe and critical COVID-19, ROC curves were drawn. For diagnosis of the severe and critical COVID-19, the area under ROC curve of IL-6 was the largest among all cytokines; the preoperative IL-6 concentration of 9.16 pg/ml was the optimal cutoff value (sensitivity = 70 (56.8–81.1)%, specificity = 82.8 (73.2–90.0)%), the positive predictive values = 73.3(60.3–84.5)%, the negative predictive values = 80.0(70.2–87.7)% (Figure 6 and Supplementary Table 6). The combined diagnosis model was established by logistic regression analysis, the area under ROC curve of combined cytokines was 0.86(0.79–0.91), the sensitivity = 80.0(67.7–89.2)%, the specificity = 75.9(65.5–84.4)%, the positive predictive values = 69.6(57.3–80.1)%, the negative predictive values = 84.6(74.7–91.8)% (Figure 6 and Supplementary Table 6).

## Potential markers predictive of critical COVID-19

**Figure 7.**
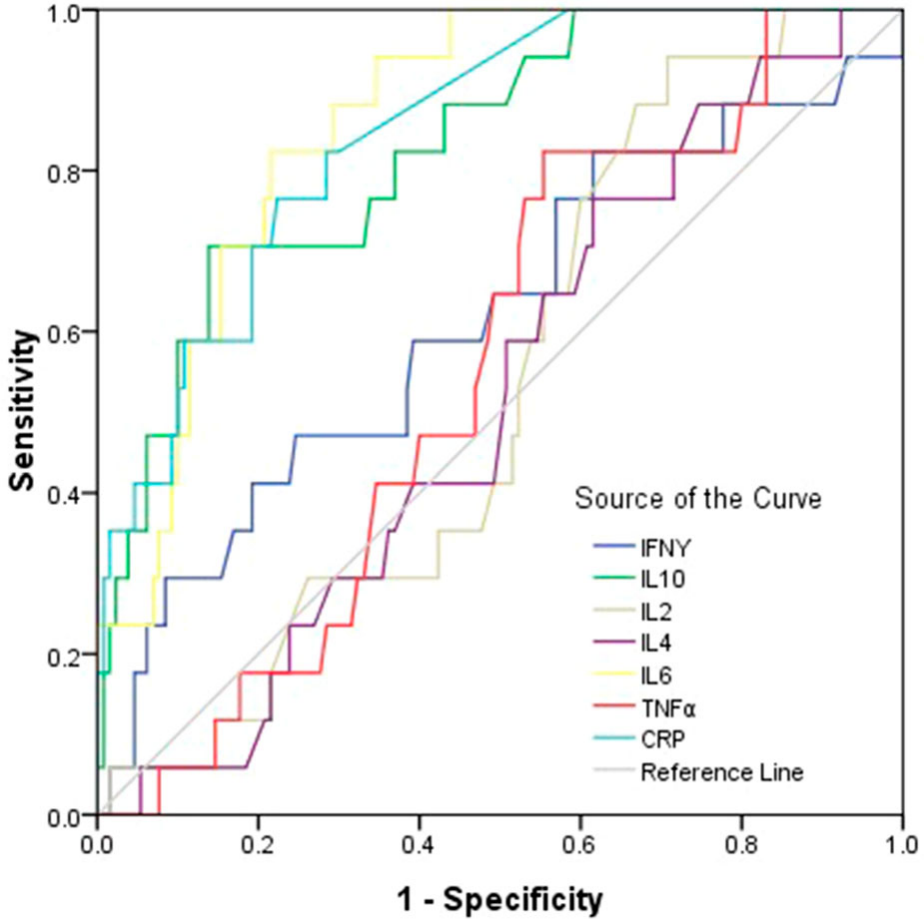
ROC curve for diagnosis of severe and critical patients with COVID-19. Univariate logistic regression analysis was used to identify the critical patients from moderate, severe COVID-19 patients and controls. Performance of ROC curves of TNF-α, IFN-γ, IL-2, IL-4, IL-6, IL-10 and CRP for predicting critical COVID-19 patients.

The combined diagnosis model was established by logistic regression analysis, results showed that combined diagnosis could not improve the diagnostic efficiency of the critical COVID-19 (Figure 7 and Supplementary Table 7). To reassure the diagnostic value of cytokines in patients with critical COVID-19, ROC curves were drawn. For diagnosis of the critical COVID-19, the area under ROC curve of IL-6 was the largest among all cytokines; the preoperative IL-6 concentration of 9.16 pg/ml was the optimal cutoff value (sensitivity = 82.4 (56.6–96.0)%, specificity = 78.5 (70.4–985.2)%), the positive predictive values = 33.3(19.6–49.5)%, the negative predictive values = 97.1(91.1–99.4)% (Figure 7 and Supplementary Table 7).

Importantly, in this cohort, one moderate patient gets worsened and eventually died after 14 days hospitalization. The symptoms of the patient was considered moderate when admitted, but the initial blood measurements revealed high IL-6 and IL-10 levels (as IFN-γ 2.12 pg/ml, TNF-α 3.79 pg/ml, IL-2 3.86 pg/ml, IL-4 3.67 pg/ml, IL-6 24.64 pg/ml, IL-10 25.66 pg/ml and CRP 19.3 mg/L). Despite the small number of such case during our study, it nevertheless highlights the importance of IL-6 and IL-10 as COVID-19 disease severity predictors.

## Discussion

The outbreak of COVID-19 was declared as a pandemic by WHO, with a crude mortality rate of about 2.3% [15]. While the most confirmed cases are considered mild, involving mostly cold-like symptoms to mild pneumonia, 14% of confirmed cases have been “severe,” involving serious pneumonia and shortness of breath. Another 5% of patients confirmed to have the disease developed respiratory failure, septic shock, and/or multi-organ failure – namely “critical cases” potentially resulting in death. As the number of COVID-19 patients is increasing dramatically worldwide and treatment in intensive care units (ICU) has become a major challenge, the early recognition of severe forms of COVID-19 is essential for timely triaging of patients. In this study, we provided evidences that inflammation reflected by cytokine storms and CRP in COVID-19 patients could have contributed to disease worsening. Given the high levels of cytokines induced by SARS-CoV-2, treatment to reduce inflammation-related lung damage is critical. More importantly, our analysis on expression and predictive values of IL-10 and IL-6 is the first proof-of-concept that those two markers should be preferentially evaluated for early diagnosis of patients with more severe disease, especially under the heavy burden of medical care in each affected hospital.

For reasons that aren’t entirely clear, some people – especially the elderly and sick – may have dysfunctional immune systems that fail to keep the response to certain pathogens in check. This could cause an uncontrolled immune response, triggering an overproduction of immune cells and their signalling molecules and leading to a cytokine storm often associated with a flood of immune cells into the lung. Similar to SARS-CoV and MERS-CoV, SARS-CoV-2 induces excessive and aberrant non-effective host immune responses that are associated with severe lung pathology, leading to death [16–18]. Like patients with SARS-CoV and MERS-CoV, some patients with 2019-nCoV develop acute respiratory distress syndrome with characteristic pulmonary ground glass changes on imaging. In most moribund patients, SARS-CoV-2 infection is also associated with a cytokine storm, which is characterized by increased plasma concentrations of different cytokines [16–19].

The pathogenesis of COVID-19 involves a potent inflammatory response, involving a complex group of mediators including IL-6 and IL-10. These pleiotropic cytokines are produced at sites of tissue inflammation and released into the circulation by a variety of different cell types, including macrophages, lymphocytes, endothelial cells, epithelial cells and fibroblasts during sepsis and acute organ injuries [20]. In influenza infection, IL-10 is highly abundant, especially during the adaptive immune response [21]. IL-6 acts as a major pro-inflammatory mediator for the induction of the acute phase response [22], leading to a wide range of local and systemic changes including fever, leucocytes recruitment and activation and hemodynamic effects. Considering the key role of IL-6 in mediating the acute phase response, its value as a prognostic biomarker in sepsis and various acute organ injuries has been extensively investigated in clinical and experimental studies. Plasma and/or bronchoalveolar levels of IL-6 have been identified as early biomarkers of lung injury and predictive factors of prolonged mechanical ventilation, organ dysfunctions, morbidity and mortality in lung diseases [23,24].

IL-6 contributes to host defense against infections and tissue injuries. However, exaggerated, excessive synthesis of IL-6 while fighting SARS-CoV-2 leads to an acute severe systemic inflammatory response cytokine storm. Remarkable beneficial effects of IL-6 blockade therapy using a humanized anti-IL-6 receptor antibody, tocilizumab was recently observed in patients with cytokine release syndrome complicated by T-cell engaged therapy [25,26]. Based on our current study, we propose the possibility that IL-6 blockade may constitute a novel therapeutic strategy for severe and critical COVID-19 patients.

However, any intervention to reduce inflammation may affect negatively on viral clearance. Thus, more clinic studies need to be conducted to find the right balance between immune activation and inflammatory inhibition.

## Supplementary Material

Supplemental Material
