# Supplementary material for: Profiling serum cytokines in COVID-19 patients reveals IL-6 and IL-10 are disease severity predictors
Source: Emerg Microbes Infect. 2020 May 31;9(1):1123–30. doi: 10.1080/22221751.2020.1770129 (PMC7473317; doi:10.1080/22221751.2020.1770129)
Supplement: Supplemental Material [file TEMI_A_1770129_SM3242.docx]

**Profiling Serum Cytokines in COVID-19 Patients Reveals IL6 and IL10 are Disease Severity Predictors**

Huan Han^1#^, Qingfeng Ma^2#^, Cong Li^3#^, Rui Liu^1^, Li Zhao^3^, Wei Wang^4^, Pingan Zhang^1^, Xinghui Liu^5^, Guosheng Gao^6^, Fang Liu^7^, Yingan Jiang^8^, Xaioming Cheng^9^, Chengliang Zhu^1*^, Yuchen Xia^3*^

1. Department of Clinical Laboratory, Renmin Hospital of Wuhan University, Wuhan 430060, Hubei, China
2. Department of Clinical Laboratory, Liyuan Hospital, Tongji Medical College, Huazhong University of Science and Technology, Wuhan 430077, Hubei, China
3. State Key Laboratory of Virology and Hubei Province Key Laboratory of Allergy and Immunology, School of Basic Medical Sciences, Wuhan University, Wuhan, China.
4. Department of Clinical Laboratory, Puai Hospital, Tongji Medical College, Huazhong University of Science and Technology, Wuhan 430033, Hubei, China
5. Department of Clinical Laboratory, Shanghai Gongli Hospital, the Second Military Medical University, Pudong New Area 200135, Shanghai, China
6. Department of Clinical Laboratory, HwaMei Hospital, University of Chinese Academy of Sciences, Ningbo 315010, Zhejiang, China
7. State Key Laboratory of Virology, College of Life Sciences, Wuhan University, Wuhan 430072, Hubei, China
8. Department of Infectious Diseases, Renmin Hospital, Wuhan University, Wuhan, China
9. Liver Diseases Branch, National Institute of Diabetes and Digestive and Kidney Diseases, National Institutes of Health, Bethesda, Maryland, 20892, USA

# These authors have contributed equally to this work.

*Corresponding to:

Prof. Yuchen Xia, State Key Laboratory of Virology and Hubei Province Key Laboratory of Allergy and Immunology, School of Basic Medical Sciences, Wuhan University, Wuhan, China. Email: yuchenxia@whu.edu.cn

Or

Chengliang Zhu, M.D., Department of Clinical Laboratory, Renmin Hospital of Wuhan University, 238 Jiefang Rd., 430060 Wuhan, People’s Republic of China. E-mail address: xinchengzhu@163.com.

**Supplementary Tables**

**Supplementary Table 1: The information of case group and control group**

| **Group** | | **Gender** | **n** | **Age (mean±SD)** | **Days of fever when admitted（median and range）** |
| --- | --- | --- | --- | --- | --- |
| Healthy control | | male | 22 | 59.8±9.7 | NA |
|  |  | female | 23 |  |  |
| COVID-19: moderate | | male | 20 | 58.3±12.6 | 14（9,18） |
|  |  | female | 22 |  |  |
| COVID-19: severe | | male | 21 | 59.3±14.4 | 10（6,15） |
|  |  | female | 22 |  |  |
| COVID-19: critical | | male | 9 | 65.1±14.4 | 10（7,11） |
|  |  | female | 8 |  |  |
|  |  |  | *χ2*=0.138 *P*>0.05 | *F*=1.567 P>0.05 | H=5.423 P=0.066 |

Chi-square tests (*χ*2 tests) were used to compare the gender ratio in different groups. Age is presented as mean ±SD by using analysis of variance (ANOVA). Days of fever when admitted were presented as median (range) for skewed distribution by Kruskal-Wallis H Test. In all tests, p value < 0.05 was defined as statistically significant. χ2 is the statistical parameter for Chi-square tests, F is the statistical parameter for analysis of variance (ANOVA) and H is the statistical parameter for Kruskal-Wallis H Test, respectively. In all tests, p value < 0.05 was defined as statistically significant.

**Supplementary Table 2: The difference of cytokines and CRP between COVID-19 patients and healthy controls**

|  | **COVID-19（n=102)** | **Controls（n=45)** | **Z** | **P** |
| --- | --- | --- | --- | --- |
| IFN-γ(pg/ml) | 3.32(2.82,4.29) | 2.73(2.43,2.99) | 3.941 | <0.001 |
| TNF-α(pg/ml) | 3.27(2.82,5.55) | 2.68(2.08,3,20) | 4.907 | <0.001 |
| IL-2(pg/ml) | 3.55(3.16,3.85) | 2.89(2.54,3.31) | 5.358 | <0.001 |
| IL-4(pg/ml) | 3.38(3.05,3.76) | 2.79(2.59,3.43) | 4.410 | <0.001 |
| IL-6(pg/ml) | 9.52(5.92,16.71) | 4.79(3.78,5.62) | 6.220 | <0.001 |
| IL-10(pg/ml) | 5.37(4.80,6.47) | 3.91(3.29,4.99) | 6.569 | <0.001 |
| CRP(mg/L) | 5.56(4.90,27.88) | 0.40(0.40,0.57) | 8.548 | <0.001 |

Date was expressed as median (range) for skewed distribution by Wilcoxon rank sum tests. In all tests, p value < 0.05 was defined as statistically significant. Z is the statistical parameter for Wilcoxon rank sum tests.

**Supplementary Table 3: The relationship between the severity of COVID-19 and cytokines and CRP**

|  | **moderate (n=42)** | **severe (n=43)** | **critical (n=17)** | **H** | **P** |
| --- | --- | --- | --- | --- | --- |
| IFN-γ(pg/ml) | 3.34(2.34,4.07) | 3.18(2.96,4.24) | 3.33(2.83,4.80) | 0.881 | 0.644 |
| TNF-α(pg/ml) | 3.18(2.85,3.41) | 4.61(2.81,14.67) | 3.16(2.99,3.72) | 7.172 | 0.028 |
| IL-2(pg/ml) | 3.40(2.91,3.82) | 3.67(3.45,3.91) | 3.32(3.15,3.81) | 7.232 | 0.027 |
| IL-4(pg/ml) | 3.36(3.06,3,67) | 3.44(2.99,3.92) | 3.27(2.92,3.71) | 1.438 | 0.487 |
| IL-6(pg/ml) | 6.23(5.55,8.96) | 10.49(6.23,20.91) | 19.48(11.28,79.14) | 26.524 | 0.000 |
| IL-10(pg/ml) | 5.05(4.59,6.14) | 5.30(4.72,6.02) | 7.02(5.30,15.63) | 11.953 | 0.003 |
| CRP(mg/L) | 4.90(4.90,15.86) | 4.90(4.90,23.50) | 34.90(8.25,125.15) | 15.352 | 0.000 |

Date was expressed as median (range) for skewed distribution by Kruskal-Wallis H Test. In all tests, p value < 0.05 was defined as statistically significant. H is the statistical parameter for Kruskal-Wallis H Test.

**Supplementary Table 4: Comparison of cytokines and CRP before and after treatment**

| **moderate** | | | | |
| --- | --- | --- | --- | --- |
|  | Before（n=29) | After（n=29) | z | P |
| IFN-γ(pg/ml) | 3.46(2.71,4.15) | 3.41(3.21,4.39) | -2.175 | 0.030 |
| TNF-α(pg/ml) | 3.21(2.92,3.68) | 3.36(3.07,4.54) | -0.638 | 0.524 |
| IL-2(pg/ml) | 3.57(2.98,3.84) | 4.00(3.76,4.40) | -3.979 | 0.000 |
| IL-4(pg/ml) | 3.43(3.09,3.74) | 3.44(3.10,4.04) | -0.900 | 0.368 |
| IL-6(pg/ml) | 6.46(5.65,9.87) | 8.02(6.12,11.67) | -1.697 | 0.090 |
| IL-10(pg/ml) | 5.54(4.85,6.21) | 5.77(5.32,6.90) | -1.763 | 0.078 |
| CRP(mg/L) | 4.90(4.90,13.70) | 4.90(4.90,4.90) | -1.053 | 0.130 |
|  | | | | |
| **severe** | | | | |
|  | Before（n=23) | After（n=23) | z | P |
| IFN-γ(pg/ml) | 3.14(2.96,4.24) | 3.31(2.95,3.48) | -0.380 | 0.704 |
| TNF-α(pg/ml) | 4.78(3.10,10.48) | 3.40(2.88,4.99) | -2.342 | 0.019 |
| IL-2(pg/ml) | 3.62(3.22,3.91) | 3.76(3.58,4.28) | -2.242 | 0.025 |
| IL-4(pg/ml) | 3.32(2.96,3.68) | 3.38(2.99,3.68) | -0.350 | 0.726 |
| IL-6(pg/ml) | 11.21(8.00,22.34) | 7.28(4.88,13.54) | -2.464 | 0.014 |
| IL-10(pg/ml) | 5.30(4,.67,6.45) | 5.91(5.38,6.93) | -2.008 | 0.045 |
| CRP(mg/L) | 10.40(4.90,28.50) | 4.90(4.90,10.40) | -2.783 | 0.005 |
|  | | | | |
| **critical** | | | | |
|  | Before（n=14) | After（n=14) | z | P |
| IFN-γ(pg/ml) | 3.18(2.72,4.18) | 3.2(2.83,3.57) | -0.245 | 0.807 |
| TNF-α(pg/ml) | 3.23(2.87,3.68) | 3.13(2.39,6.70) | -0.282 | 0.778 |
| IL-2(pg/ml) | 3.32(3.16,3.60) | 3.74(3.48,4.01) | -2.354 | 0.019 |
| IL-4(pg/ml) | 3.17(2.83,3.69) | 3.16(2.78,3.56) | -0.534 | 0.594 |
| IL-6(pg/ml) | 19.48(10.85,53.55) | 15.66(8.31,23.74) | -0.910 | 0.363 |
| IL-10(pg/ml) | 6.80(5.33,15.12) | 7.17(6.12,9.52) | -0.534 | 0.594 |
| CRP(mg/L) | 32.80(5.80,86.43) | 4.90(6.30,44.93) | -2.432 | 0.015 |

Date was expressed as median (range) for Wilcoxon signed-rank test.

**Supplementary Table 5. Area under the curve of cytokines and CRP**

| **Area Under the Curve** | | | | | |
| --- | --- | --- | --- | --- | --- |
| Test Result Variable(s) | Area | Std. Error^a^ | Asymptotic Sig.^b^ | Asymptotic 95% Confidence Interval | |
|  |  |  |  | Lower Bound | Upper Bound |
| IFN-γ | .704 | .044 | .000 | .617 | .791 |
| IL-10 | .841 | .034 | .000 | .773 | .908 |
| IL-2 | .778 | .045 | .000 | .689 | .867 |
| IL-4 | .729 | .051 | .000 | .628 | .829 |
| IL-6 | .822 | .038 | .000 | .748 | .896 |
| TNF-α | .754 | .043 | .000 | .671 | .838 |
| CRP | .955 | .017 | .000 | .921 | .989 |
| The test result variable(s): IFN-γ, IL-10, IL-2, IL-4, TNF-α, CRP has at least one tie between the positive actual state group and the negative actual state group. Statistics may be biased. | | | | | |
| a. Under the nonparametric assumption | | | |  |  |
| b. Null hypothesis: true area = 0.5 | | | |  |  |

**Supplementary Table 6. Area under the curve for diagnosis of severe and critical patients with COVID-19**

| **Area Under the Curve** | | | | | |
| --- | --- | --- | --- | --- | --- |
| Test Result Variable(s) | Area | Std. Error^a^ | Asymptotic Sig.^b^ | Asymptotic 95% Confidence Interval | |
|  |  |  |  | Lower Bound | Upper Bound |
| IFN-γ | .654 | .046 | .001 | .564 | .745 |
| IL-10 | .725 | .041 | .000 | .644 | .805 |
| IL-2 | .716 | .042 | .000 | .635 | .798 |
| IL-4 | .627 | .046 | .009 | .537 | .717 |
| IL-6 | .826 | .034 | .000 | .759 | .893 |
| TNF-α | .711 | .044 | .000 | .624 | .798 |
| CRP | .802 | .036 | .000 | .731 | .873 |
| The test result variable(s): IFN-γ, IL-10, IL-2, IL-4, IL-6, TNF-α, CRP has at least one tie between the positive actual state group and the negative actual state group. Statistics may be biased. | | | | | |
| a. Under the nonparametric assumption | | | |  |  |
| b. Null hypothesis: true area = 0.5 | | | |  |  |

**Supplementary Table 7. Area under the curve for diagnosis of severe and critical patients with COVID-19**

| **Area Under the Curve** | | | | | |
| --- | --- | --- | --- | --- | --- |
| Test Result Variable(s) | Area | Std. Error^a^ | Asymptotic Sig.^b^ | Asymptotic 95% Confidence Interval | |
|  |  |  |  | Lower Bound | Upper Bound |
| IFN-γ | .614 | .078 | .127 | .461 | .768 |
| IL-10 | .827 | .050 | .000 | .730 | .925 |
| IL-2 | .534 | .061 | .645 | .414 | .655 |
| IL-4 | .519 | .064 | .804 | .393 | .644 |
| IL-6 | .860 | .036 | .000 | .789 | .932 |
| TNF-α | .555 | .062 | .464 | .434 | .676 |
| CRP | .848 | .044 | .000 | .762 | .933 |
| The test result variable(s): IFN-γ, IL-10, IL-2, IL-4, TNF-α, CRP has at least one tie between the positive actual state group and the negative actual state group. Statistics may be biased. | | | | | |
| a. Under the nonparametric assumption | | | |  |  |
| b. Null hypothesis: true area = 0.5 | | | |  |  |

**Supplementary Figures**


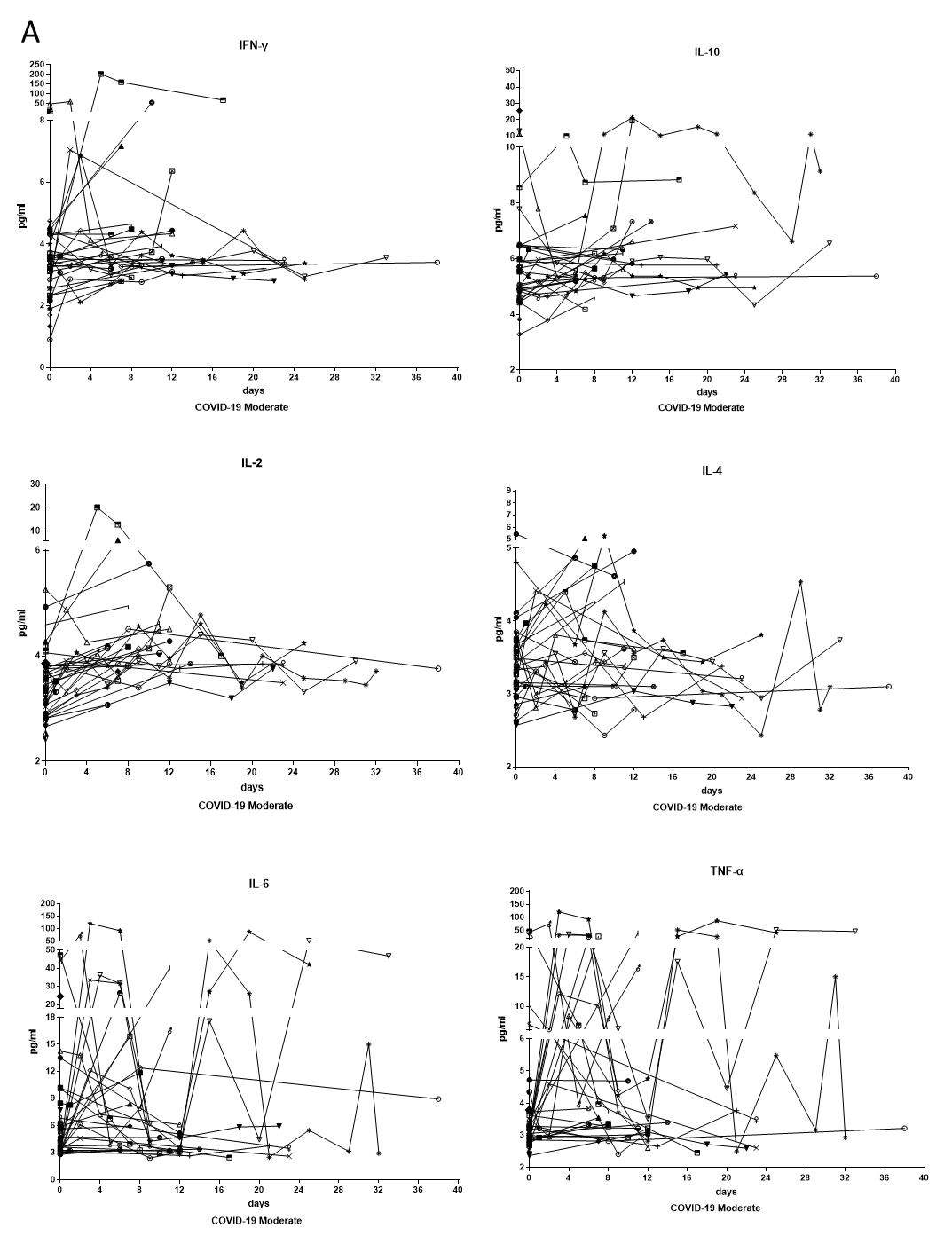


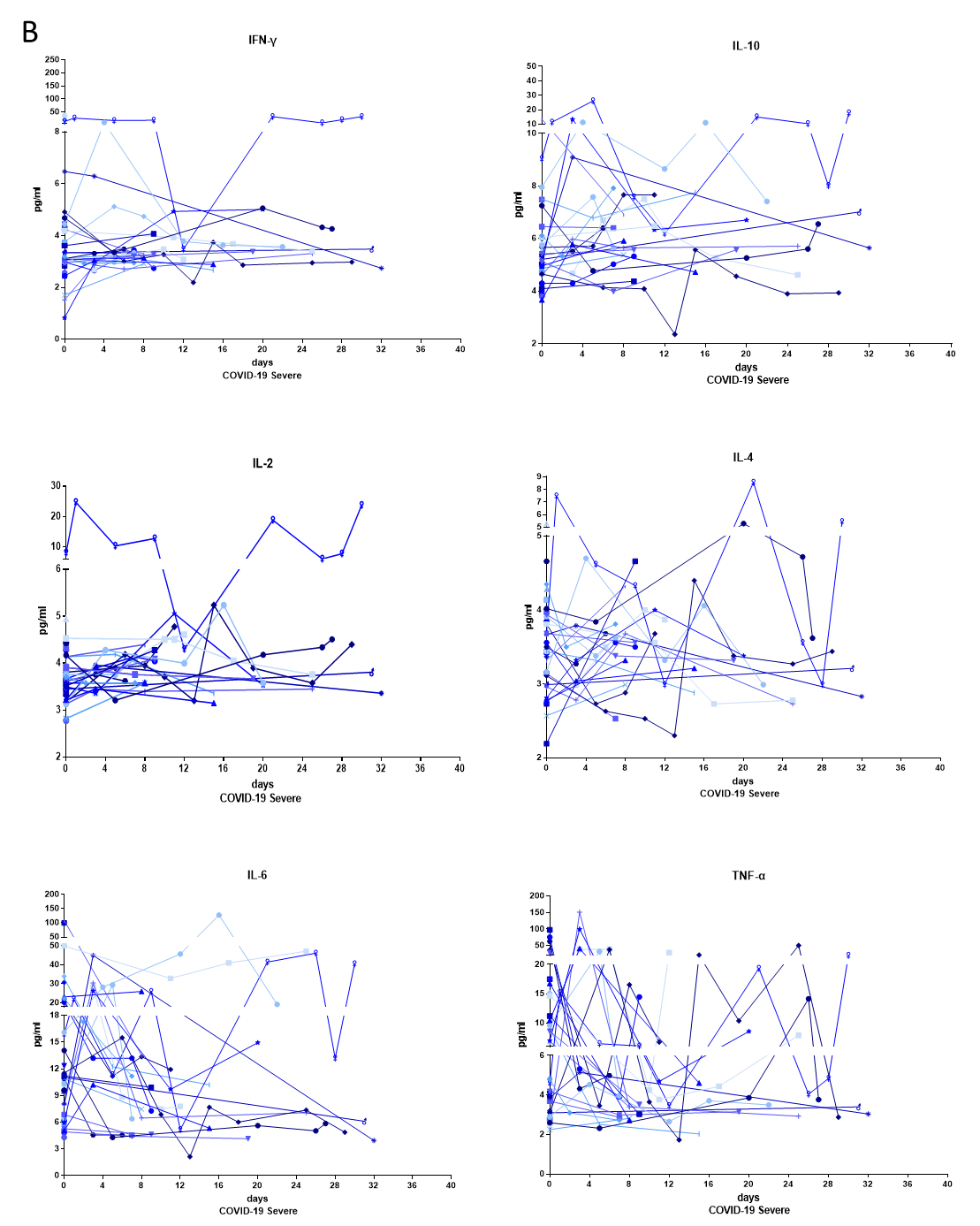


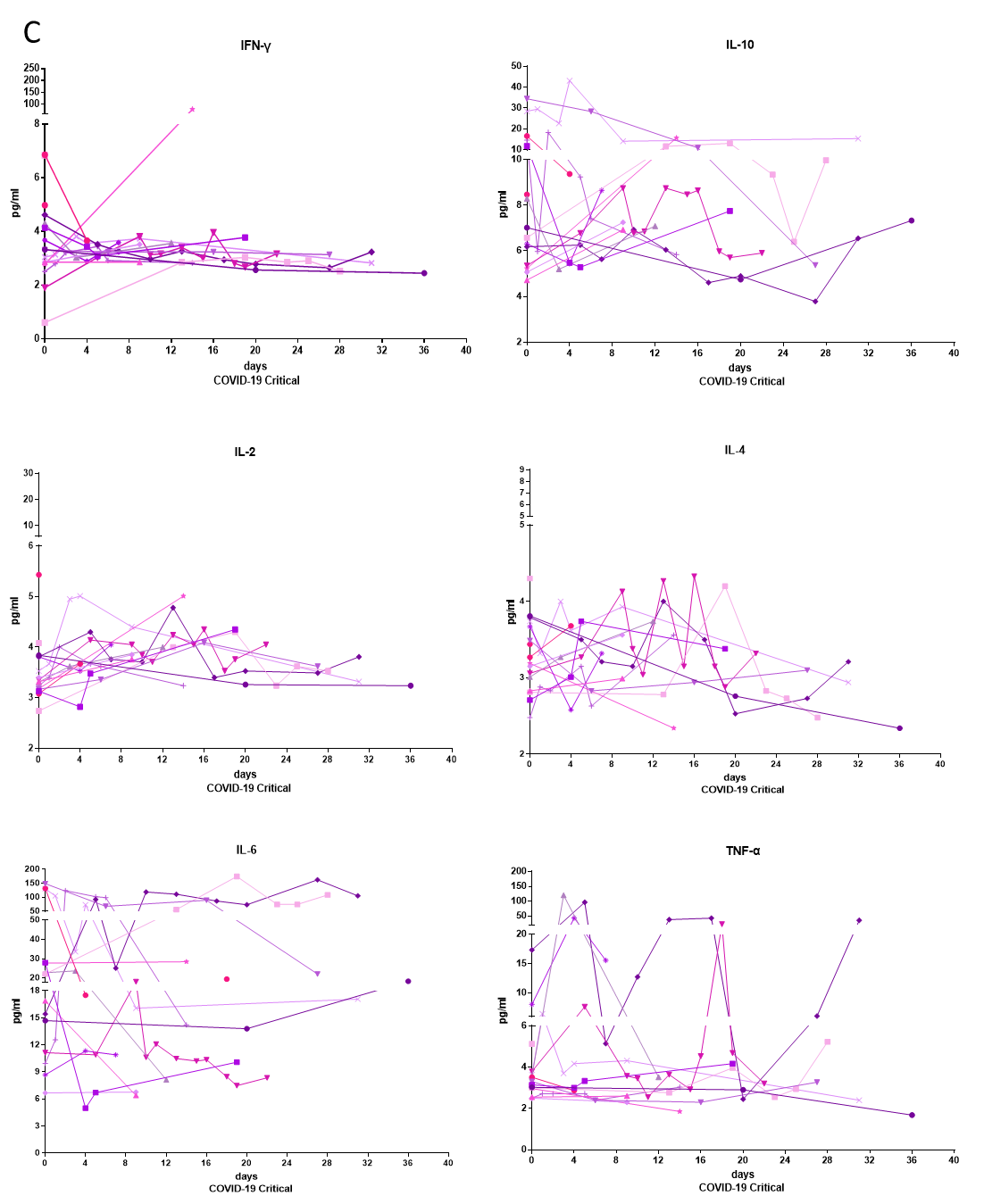


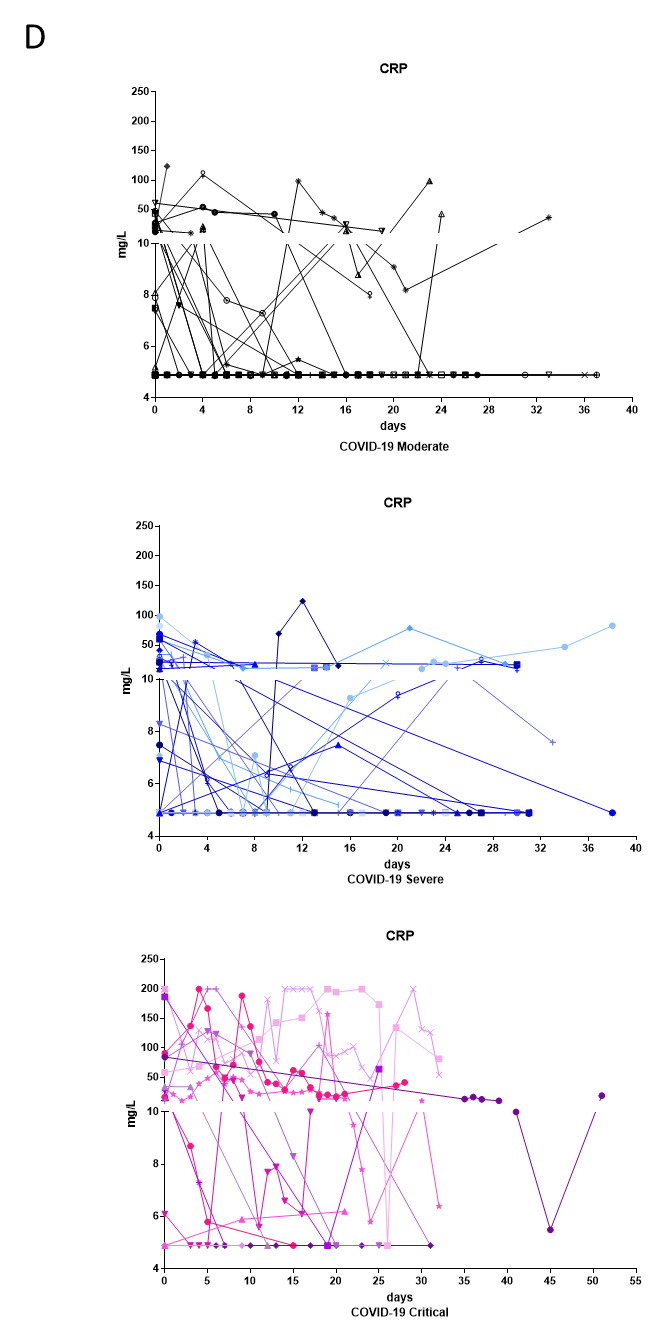


**Supplementary Figure 1: The kinetics of cytokines and CRP in COVID-19 patients during hospitalization**

The serum cytokines levels of moderate (A), severe (B) and critical (C) patients and CRP (D) level from each patient during hospitalization were presented. The x-axis represents days after admission.


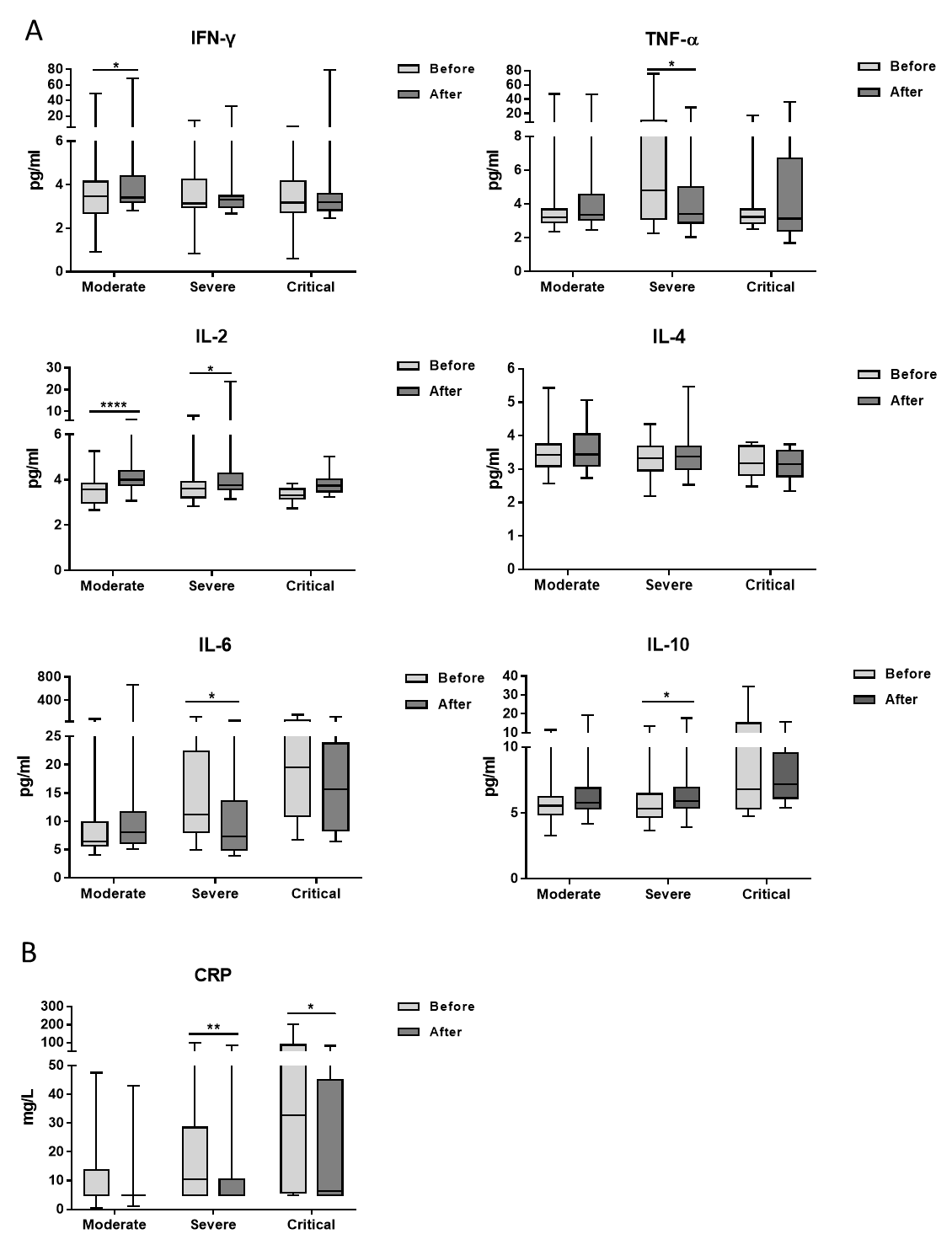


**Supplementary Figure 2: The comparison of cytokines and CRP in COVID-19 patients before and after treatment**

The serum cytokines (A) and CRP (B) levels of moderate, severe and critical patients before and after treatments were compared by Wilcoxon signed-rank test. *P ≤ 0.05, **P ≤ 0.01, ***P ≤ 0.001, **** P ≤ 0.0001.
